# Safety of Clomiphene Citrate and Its Impact on Serum Tumor Markers in Hypogonadal Testicular Cancer Patients After Chemotherapy

**DOI:** 10.7759/cureus.98741

**Published:** 2025-12-08

**Authors:** James Wren, Brian Myre, Joshua A Halpern, Evan Panken, Nelson E Bennett, Robert E Brannigan

**Affiliations:** 1 Urology, Beth Israel Deaconess Medical Center, Harvard Medical School, Harvard University, Boston, USA; 2 Oncology, Duly Health and Care, Chicago, USA; 3 Urology, Feinberg School of Medicine, Northwestern University, Chicago, USA

**Keywords:** clomiphene citrate, hypogonadism, male fertility, serum tumor markers, testicular cancer

## Abstract

Introduction

Clomiphene citrate (CC) is used to treat secondary hypogonadism. This study aimed to assess the impact of CC on hypogonadal testicular cancer survivors' serum testosterone, luteinizing hormone (LH), and beta-human chorionic gonadotropin (beta-HCG) levels.

Methods

Following institutional review board (IRB) approval, we performed a retrospective chart analysis of all patients who had undergone a radical orchiectomy from 2004 to 2022. Inclusion criteria were males >18 years of age who had undergone a radical orchiectomy and chemotherapy for testicular cancer, who were subsequently treated with CC due to symptomatic hypogonadism (serum testosterone < 300 ng/dL). Mean serum testosterone levels were compared using a paired, two-tailed t-test. Mean serum LH and beta-HCG levels were compared using a one-tailed, two-sample t-test assuming equal variance.

Results

In total, 476 radical orchiectomies were performed, with 10 patients meeting the inclusion criteria. CC was associated with a 273 ng/dL mean increase in serum testosterone (215-486 ng/dL). The mean follow-up after commencing CC therapy was 52.5 months. No side effects or evidence of disease recurrence occurred. No false-positive elevations in beta-HCG tumor marker levels related to the CC-induced rise in serum LH levels occurred.

Conclusion

This is the first study to assess the use of CC in hypogonadal testicular cancer survivors following chemotherapy. CC was associated with an increase in serum testosterone levels with no associated side effects, false-positive elevation of beta-HCG tumor marker levels, or disease recurrence.

## Introduction

Testicular cancer is the most common cancer in males aged 15-39 and has an associated cure rate of >95%. Despite the successful treatment of testicular cancer following radical orchiectomy and chemotherapy, 38% of males are rendered hypogonadal [1,2]. Hypogonadism is the constellation of a biochemically low testosterone level (<300 ng/dL) with associated symptoms of fatigue, low mood, low libido, decreased erectile function, and lean muscle mass or an increase in central adiposity [3]. Hypogonadism is commonly treated with exogenous testosterone replacement therapy (TRT); however, in males interested in future fertility, this is contraindicated due to the negative feedback inhibition on the hypothalamus-pituitary-gonadal (HPG) axis. This inhibition suppresses luteinizing hormone (LH) and follicle-stimulating hormone (FSH) release, which results in a decrease in endogenous testosterone production and spermatogenic failure [4].

Clomiphene citrate (CC) is an oral selective estrogen receptor modulator (SERM) that has been used in an off-label fashion to treat secondary hypogonadism in males interested in future fertility since the 1960s [5]. CC increases the release of LH and FSH from the anterior pituitary, resulting in increased endogenous testosterone production, while preserving spermatogenesis [6]. Despite being an inexpensive oral alternative to testosterone replacement therapy, it is not commonly used to treat hypogonadism in testicular cancer survivors following chemotherapy [7].

One potential reason for CC’s limited use is that it is mainly prescribed by andrologists who may not routinely be involved in a patient's care. A second reason is the unsubstantiated and hypothetical concern that CC, by virtue of its intended increase in LH, might inadvertently result in a false-positive elevation in the testicular cancer tumor marker, beta-human chorionic gonadotrophin (beta-HCG). LH and beta-HCG share the same beta-subunit, leading to the possibility that a CC-induced increase in LH could contribute to a false-positive beta-HCG elevation [8]. A third reason is that the hypogonadism resulting from testicular dysgenesis (orchiectomy and chemotherapy) may be primary and not a secondary hypogonadism, in which case CC would be ineffective.

Our study aimed to assess whether CC could be used in testicular cancer survivors with symptomatic secondary hypogonadism to normalize testosterone levels without resulting in a false-positive elevation in beta-HCG tumor marker levels. 

This article was previously presented at the American Urological Association (AUA) meeting (September 2021). 

## Materials and methods

Study design**

Following institutional review board (IRB) approval, we queried the Northwestern Enterprise Data Warehouse to identify all patients diagnosed with testicular germ cell tumors following a radical orchiectomy between January 2004 and January 2021. Through a retrospective chart analysis, we included all males >18 years of age with a history of both radical orchiectomy and chemotherapy for testicular germ cells tumors, who developed symptomatic secondary hypogonadism (testosterone < 300 ng/dL on two early morning samples and an LH level < 10 IU/L), who were treated with oral CC (25 mg or 50 mg daily). Symptomatic hypogonadism required an initial symptom score of 3 or more on the Androgen Deficiency of Aging Male (ADAM) questionnaire. Follow-up post-treatment scores were obtained with routine surveillance outpatient follow-up visits. Exclusion criteria included those patients with testicular germ cell tumors who did not undergo treatment with chemotherapy, those patients who were treated with other forms of testosterone replacement therapy (intramuscular or subcutaneous injections, transdermal, and pellets), and those patients who underwent radiation therapy for seminoma. Patients treated with CC for <30 days or patients who were rendered castrate following removal of a solitary testicle were excluded. Patients who were diagnosed with a prolactinoma or who did not have baseline LH and beta-HCG levels available were also excluded.

Primary outcome and statistical analysis 

The primary outcome was the serum testosterone level following CC therapy. The secondary outcomes were serum LH and beta-HCG levels following CC therapy. Descriptive statistics were utilized to characterize patient demographics, comorbidities, hormonal and tumor marker profiles (LH, testosterone, beta-HCG), CC dosage, testicular volume, medication side effects, chemotherapy regimen, testicular germ cell pathology, and tumor stage. Successful treatment of hypogonadism included symptomatic improvement associated with normalization of biochemical testosterone level (testosterone 450-600 ng/dL). Serum beta-HCG levels were tested using electro-chemiluminescent immunoassay (beta-HCG, normal <5 IU/L). Mean testosterone levels were compared using a paired, two-tailed t-test. LH and beta-HCG levels were compared using a one-tailed, two-sample t-test assuming equal variance. All analyses assumed a 5% level of significance. Analyses were performed using R: a language and environment for statistical computing (Version 3.3.2; R Foundation for Statistical Computing, Vienna, Austria).

## Results

In total, 476 radical orchiectomies were performed, with 10 patients meeting the inclusion criteria (Table 1). Nine (90%) patients elected CC therapy due to a possible interest in future fertility. The remaining patient was not interested in fertility and elected to take CC due to a lack of interest in the exogenous testosterone preparations.

**Table 1:** Patient demographics

Demographics		
Age (years)	Mean (SD)	34.2 (5)
Pathology	Seminoma	N = 5
	Non-seminoma	N = 5
Chemotherapy	Bleomycin, etoposide, cisplatin x 1	N = 1
	Bleomycin, etoposide, cisplatin x 3	N = 7
	Etoposide, cisplatin x 4	N = 1
	Carboplatin	N = 1
Stage	1	N = 3
	2	N = 5
	3	N = 2
Follow-up	Mean (SD)	53.6 (37.4)

**Figure 1:**
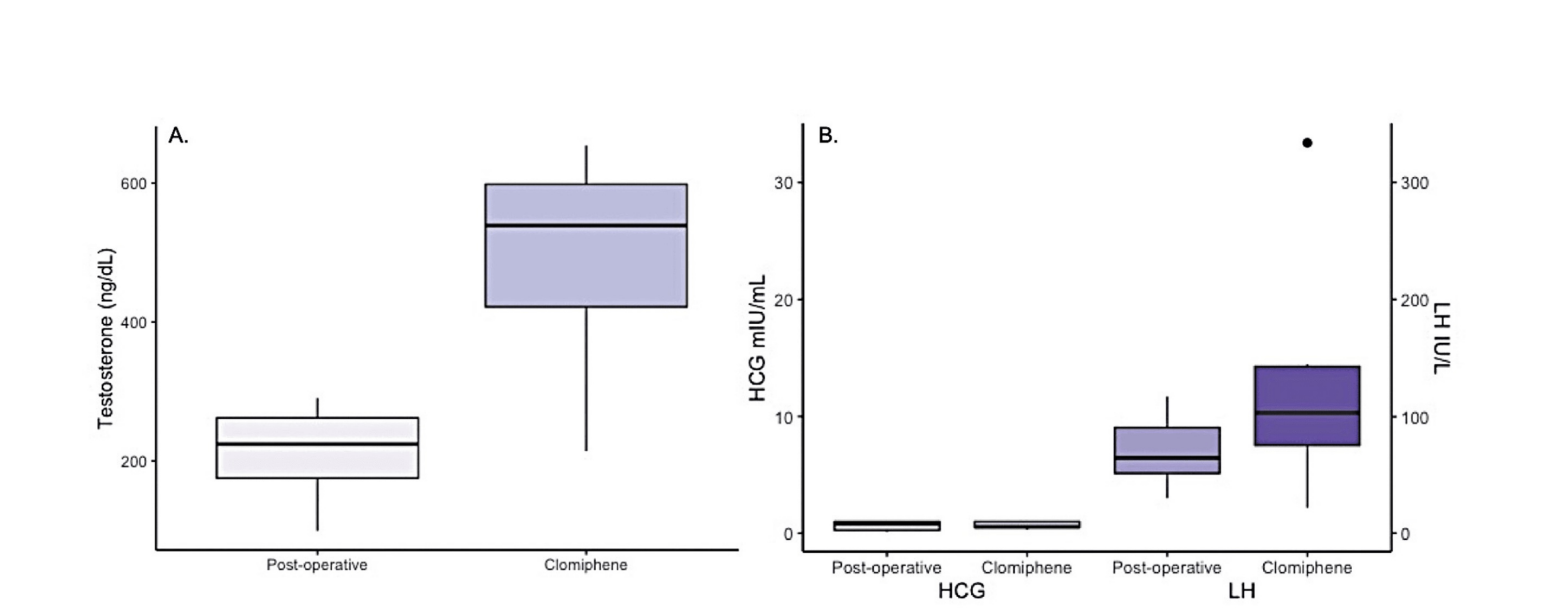
(A) Serum testosterone levels post-radical orchiectomy and chemotherapy (post-operative) and after treatment with clomiphene (p < 0.001). (B) Serum HCG and LH levels before and after treatment with clomiphene (p = 0.05) HCG: human chorionic gonadotropin, LH: luteinizing hormone.

CC was associated with a 273 ng/dL mean increase in serum testosterone levels (215-486 ng/dL, p < 0.001) in post-chemotherapy patients (Figure 1A). Eight (80%) patients required 25 mg daily dosing of CC, while 2 (20%) patients required 50 mg daily to achieve eugonadal serum testosterone levels. The mean follow-up after commencing CC was 53.6 months ± 37.4 (SD). During the follow-up period, no side effects to CC were reported, and no patients demonstrated evidence of disease recurrence. No false-positive elevation of beta-HCG tumor markers related to CC-induced LH increase occurred (7.0-13.8 IU/L, p = 0.05) (Figure 1B). In all patients, beta-HCG tumor markers remained undetectable (<1.0) (Figure 1B). The only comorbidity identified in the cohort was one patient with incidental contralateral testicular microlithiasis identified at the time of the initial scrotal ultrasound. 

## Discussion

Advances in the management of testicular cancer represent one of the major successes of modern medicine and surgery [9]. Prior to the advent of cisplatin-based chemotherapy, a diagnosis of testicular cancer was associated with a 70% overall chance of mortality. Following advances in chemotherapeutic regimens and surgical techniques, there now exists an 80% chance of survival in patients presenting with metastatic disease [9]. As a result of the success with primary endpoints of overall and cancer-specific survival, secondary endpoints in survivorship studies have emerged, which report the increasing importance that male cancer survivors place on quality-of-life factors, including future paternity [10,11].

Given the high prevalence of hypogonadism in young testicular cancer survivors (38%) and the importance placed by patients on future paternity, our study assessed whether fertility-preserving CC could be used to treat secondary hypogonadism following radical orchiectomy and chemotherapy. Our study identified 10 patients who were successfully treated with CC, while avoiding the more commonly used exogenous testosterone therapy preparations (transdermal, injectables, subcutaneous pellets, intranasal, and buccal) that negatively impact spermatogenesis [12-15]. Patients in our cohort tolerated CC and reported no side effects. Two patients who were initially started on a dose of 50 mg daily had to decrease their dosing to 25 mg daily due to supratherapeutic serum testosterone levels (>600 ng/dL) of 822 and 836 ng/dL, based on the AUA testosterone deficiency guidelines [3]. Although side effects were not reported in our cohort, previous studies have reported side effects in 8% of patients taking CC [16]. These include changes in mood, headaches, vision disturbance, flushing, and elevated estradiol-induced breast tenderness or gynecomastia [3,7,16]. Elevations of hematocrit, lipids, liver function tests, and PSA (which are commonly seen in exogenous testosterone preparations) are uncommonly observed with CC use; however, an increase in estradiol level is regularly seen [7,17-19]. 

The theoretical concern regarding CC use that we sought to investigate during this study was whether CC resulted in false-positive elevations in beta-HCG tumor marker levels in testicular cancer patients during surveillance. This concern was based on previously reported biochemical cross-reactivity of LH and beta-HCG levels during laboratory analysis [8]. However, this was during the era of radioimmunoassay (RIA) technology, which had low specificity for beta-HCG [8]. In one study, 41% of cured non-seminomatous testicular germ cell tumor patients were found to have false-positive beta-HCG elevations due to cross-reactivity with LH [20]. Current advanced beta-HCG diagnostic testing uses an electro-chemiluminescent immunoassay, which has a much lower cross-reactivity rate of 0.12% with LH [21]. However, the risk that a CC-induced LH elevation could translate into a false-positive beta-HCG elevation, resulting in a misdiagnosis of cancer recurrence, would ultimately prevent CC’s use in this patient population. Our study did not find a CC-associated increase in beta-HCG levels despite the increase in LH levels. There was also no evidence of disease recurrence on surveillance imaging in any patients during the mean follow-up of 53.6 months.

Limitations to the study include its small retrospective nature, heterogeneous pathology and chemotherapy regimens, and varied disease staging. Other limitations include the non-specific hypogonadal symptoms, which could have been attributed to the post-chemotherapy effect in a young, concerned cancer patient population. Ideally, patients would have undergone interval semen analysis prior to commencing CC, followed by serial semen analysis during CC treatment to assess changes in semen parameters. However, the well-recognized unpredictable spermatogenic response patients have to chemotherapy would have confounded these results [21].

## Conclusions

This is the first study to assess the use of CC in hypogonadal testicular germ cell survivors following radical orchiectomy and chemotherapy. CC was associated with an increase in serum testosterone levels with no reported side effects. There was no evidence of disease recurrence and no associated false-positive elevation in beta-HCG tumor markers. Further studies are needed to define the role of CC use in testicular cancer survivors with secondary symptomatic hypogonadism.
